# Mapping wader biodiversity along the East Asian—Australasian flyway

**DOI:** 10.1371/journal.pone.0210552

**Published:** 2019-01-25

**Authors:** Jia Li, Alice C. Hughes, David Dudgeon

**Affiliations:** 1 School of Biological Sciences, The University of Hong Kong, Hong Kong SAR, China; 2 Centre for Integrative Conservation, Xishuangbanna Tropical Botanic Garden, Chinese Academy of Sciences, Yunnan, China; MARE – Marine and Environmental Sciences Centre, PORTUGAL

## Abstract

**Background and goal:**

The study is conducted to facilitate conservation of migratory wader species along the East Asian-Australasian Flyway, particularly to 1) Identify hotspots of wader species richness along the flyway and effectively map how these might change between breeding, non-breeding and migratory phases; 2) Determine if the existing network of protected areas (PA) is sufficient to effectively conserve wader biodiversity hotspots along the EAAF; 3) Assess how species distribution models can provide complementary distribution estimates to existing BirdLife range maps.

**Methods:**

We use a species distribution modelling (SDM) approach (MaxEnt) to develop temporally explicit individual range maps of 57 migratory wader species across their annual cycle, including breeding, non-breeding and migratory phases, which in turn provide the first biodiversity hotspot map of migratory waders along the EAAF for each of these phases. We assess the protected area coverage during each migration period, and analyse the dominant environmental drivers of distributions for each period. Additionally, we compare model hotspots to those existing range maps of the same species obtained from the BirdLife Internationals’ database.

**Results:**

Our model results indicate an overall higher and a spatially different species richness pattern compared to that derived from a wader biodiversity hotspot map based on BirdLife range maps. Field observation records from the eBird database for our 57 study species confirm many of the hotspots revealed by model outputs (especially within the Yellow Sea coastal region), suggesting that current richness of the EAAF may have been underestimated and certain hotspots overlooked. Less than 10% of the terrestrial zones area (inland and coastal) which support waders are protected and, only 5% of areas with the highest 10% species richness is protected.

**Main conclusions:**

The study results suggest the need for new areas for migratory wader research and conservation priorities including Yellow Sea region and Russian far-East. It also suggests a need to increase the coverage and percentage of current PA network to achieve Aichi Target 11 for Flyway countries, including giving stronger consideration to the temporal dynamics of wader migration.

## Introduction

Globally, approximately 20% of bird species migrate on an annual basis, typically following set migratory routes [1]. The East Asian-Australasian Flyway (EAAF) is one such flyway stretching from Alaska and North Eastern Russia, to Australia and New Zealand [2]. Some long-distance migrants within the flyway breed in the high arctic and spend their non-breeding season across Australia and New Zealand. In contrast medium/short distance migrants breed in their northern or southern portions of their ranges and spend the non-breeding season closer to the equator. The EAAF provides a theoretical framework developed principally to facilitate the conservation of migratory birds in the East Asian and Australasian region [2, 3]. This flyway is used by 492 species, comprising more than 50 million migrants annually of which over 5 million are waders [4–6]. This latter group is regarded as a priority for conservation [7–10].

The continuing existence of the species that rely on this flyway are being threatened by the loss of coastal habitats and environmental degradation [11, 12], resulting in population reductions of many migrants, including at least eight species of waders [13, 14]. Decline rates of waterbird species along the EAAF have been estimated at 5–9% per year, and are among the highest reported for any ecosystem [11], and may be even higher for some species such as the critically endangered spoon-billed sandpiper (*Calidris pygmeus*). Some of the more charismatic migrants have been studied as individual species, including the black-faced spoonbill (*Platalea minor*) [15, 16], red-crowned crane (*Grus japonensis*) [17] and bar-tailed godwits (*Limosa lapponica*) [18]. However, the distribution of migrating waders along the EAAF has not previously been investigated as a group, although some recent work has involved mapping potential migratory routes of multiple species [19] and identifying key sites for conservation [20].

Given the threats facing migratory waders and their intertidal habitats, a map of wader species richness provides a useful tool to convey to decision makers of both the distribution of species richness and important sites. This research aims to enhance existing maps of species richness along the EAAF, and to supplement conservation efforts based on BirdLife range maps, through additional research outputs using species distribution modelling (SDM) and citizen science data. The BirdLife species range maps [21] represent estimates of species distributions based on existing data at the time of the assessment, supplemented with interpolation by experts [21, 22]. BirdLife range maps are widely used and provide cost-effective scientific input for developing broad-scale conservation strategies [23, 24]. However, these maps are seldom regionally verified at high spatial or temporal precision; they also show some discrepancies with current records [25, 26], limiting their application in effective conservation planning decisions [27]. SDM approaches offer an alternative way to estimate species range by combining environmental data and species occurrence records, and to explore the changing distributions and dependencies of species over time [28, 29]. SDMs can provide additional and timely scientific evidence to support conservation actions, taking account of citizen science data that can be updated and supplemented frequently [30]. Thus this study was expected to provide new insights on potential hotspots or important areas for further on-the-ground investigation by utilizing latest citizen science data and SDM techniques.

Conservation of migratory species presents different challenges to that of sedentary species [31], yet most Protected Areas (PA) are designated based on site level needs and with sedentary species in mind [32, 33]. Indeed, only 9% of migratory bird species are adequately covered by protected areas across all stages of their annual cycle in comparison with 45% PA coverage of non-migratory birds [33]. In this study, we compared our modelled distribution results of migratory birds at different stages of their migratory cycles with the current extent of the PA network along the EAAF. The specific objectives of this study were threefold. Firstly, to identify and compare hotspots of wader biodiversity derived from two mapping methodologies (expert-drawn range maps and SDM) to identify the temporal and spatial priorities for conservation along the EAAF. Secondly, to compare the model outputs to the existing network of Protected Areas (PAs) along the EAAF in order to determine its sufficiency for conserving wader biodiversity hotspots. Third, to compare the distribution estimates using different methodologies for conservation priority setting and management actions, particularly whether SDMs could provide a useful addition to the current approach based on expert assessment.

## 2. Materials and methods

In this study, we used MaxEnt (Maximum Entropy) SDM to develop range maps for 57 wader species and overlay them to produce a consolidated map of wader biodiversity hotspots along the EAAF for breeding, non-breeding and migration. We also used the range maps of the same 57 wader species available from BirdLife database [21] to produce comparative hotspot maps of wader species along the EAAF.

In this study, the term “biodiversity hotspot” refers to areas with concentrations of species richness within the study area. Our use of the term “waders”, which is commonly used interchangeably with “wading birds” or “shorebirds”, refers to Charadriiformes that are dependent on aquatic habitats, and particularly intertidal zones, during a significant part of their lives; most wade through shallow water during feeding. We focus upon waders, as a number of species have suffered significant population declines along the EAAF in recent years [13, 34].

### 2.1. MaxEnt

MaxEnt (Maximum Entropy) is a commonly-used SDM approach [29]. It has proven effective and efficient for modelling complex interactions between environmental variables and species occurrences in over 1000 published applications since its release in 2006 [29, 35]. One of the strengths of Maxent is that it does not require absence data, which is especially important for wide ranging species [36], and it can accommodate extreme values [37]. However, MaxEnt has predominantly been used to model the distribution of species that show little seasonal change in site occupancy. A static map for mobile species—especially migrants—lacks the dynamism needed to fully understand the drivers of distribution across either space or time, as limiting factors may vary during the year due to changing requirements during different phases of migration (including breeding and sedentary non-breeding phases). To achieve the same temporal resolution as in BirdLife range maps, we included a temporal component to SDM to take account of changing patterns of distribution due to migration. This allowed us to identify the environmental variables associated with such changes at different migratory stages.

The species selected were all listed as migratory waders by the EAAF Partnership (EAAFP, https://eaaflyway.net/migratory-waterbirds/) and they heavily depend on tidal flats [1]. We used the MaxEnt modelling approach combined with occurrence data from eBird (http://ebird.org/content/ebird/; [38] to generate SDM-derived range maps and overlay to produce a biodiversity hotspot map of wader species. All spatial analysis was conducted using ArcGIS 10.2 and QGIS (version 2.12), while SDM was performed with MaxEnt 3.3.3k (https://www.cs.princeton.edu/~schapire/maxent/) [29, 39].

#### 2.1.1. Species data preparation

Species point-occurrence data were downloaded from eBird and extracted using the appropriate geographical mask (EAAF). Occurrence data collected between 2010–2015 were used to reflect current species distributions, rather than potentially over-predicting species ranges by pairing former range data which may not temporally align with current conditions (e.g. due to land-use changes, and increasing extent of human settlement) and thus may no longer be suitable. A total of 217,741 records for the 57 species were used in our models (for a full list of the species see S2 Table).

We use substantial range shifts between consecutive months to delimit species distribution in detectable migration movement periods for each species, denoting northward and southward migrations in addition to breeding and sedentary non-breeding ranges (S2 Table). A fishnet was created to calculate the number of occurrence records of each species each month within each 50km x 50 km square (S1 Fig), so that the movement of each species over an annual migration cycle could be reviewed across the EAAF using the monthly point occurrence data for each species. These time periods were then used as the basis to develop seasonally explicit climate data for each phases of each species, and models developed separately using paired distribution and climate data for each temporal phase. All analyses were then carried out at 1x1km resolution.

#### 2.1.2. Environmental data preparation

To provide a realistic estimate of species distribution, SDMs for specific species must be based on environmental variables important for waders. To do this, we parameterised climatic variables over specific temporal periods when each species was “stationary” in a region. Models were developed for individual species for each migratory phase using corresponding seasonal climatic data, paired with relevant topographic data. Climate data were split according to the phases of the year when birds were stationary or migrating: non-breeding, northward migration, breeding and southward migration. Once the appropriate months within each phase had been grouped (S2 Table), the cell statistics tool was used to find the maximum, minimum, mean and variability (standard deviation) of temperature, and the mean and variability of precipitation over each movement phase for each species (generating a total of 136 different seasonal blocks based on different temporal periods for each of the four phases, with between one to four distinct phases per species). These factors have the potential to limit the ability of a species to survive and access the resources [40, 41].

In addition to temporally partitioned climate data, additional environmental data which does not vary seasonally (i.e. altitude) were used in all of the 136 environmental data sets. These “static” environmental layers included altitude (www.worldclim.org), land use (http://due.esrin.esa.int/page_globcover.php), distance to coast, distance to intertidal-flats, and distance to the nearest waterway, soil parameters (silt, clay, and sand, soil coarseness, soil organic matter and soil pH, downloaded from ISRIC 1KM global soil database) [42], and night-time lights (2013) as a proximate indicator of human settlement (http://ngdc.noaa.gov/eog/). Methods used to generate these layers are described in S1 Table. Land-use is often an important factor determining species distribution [43, 44]. The topographic factors (altitude, distance to coast and distance to intertidal flats) were used to model favourable habitat requirements (e.g. shallow water along the coasts), while the soil parameters were used to model availability of food. Soil type and organic matter content are likely to relate to prey availability and ease of capture by waders [45–47]. The relationship between night-time light and human settlement is complex and not always linear [48]. However, night-time light is strongly correlated with human activities [49] and if coupled with land use, represent a good proxy for development intensity [50]. All data used in models was at a 1km resolution.

#### 2.1.3. Modelling

Each Maxent model retained 15% species occurrence data as random test data to test the accuracy of models, and each model ran 10 replicates which were then averaged prior to reclassification. Default settings were used, as this approach is considered to be most cost-effective for maximum accuracies across multiple models without individual configuration [29]. The output maps of binary species distributions were generated based on a 10 percentile training presence threshold to delineate suitable from unsuitable areas [51]. The 10 percentile training presence threshold is one of the most commonly-used thresholds used within Maxent [52], due to its ability to accurately and conservatively predict species distributions relative to other thresholds. It has been shown to have the lowest levels of bias [53] and the highest accuracy [54] even for locally restricted and endemic species [55].

All models had an AUC (Area under Curve) range of 0.894–0.999 and a mean AUC of 0.984 (±0.014). The resulting range maps were combined to produce the wader biodiversity hotspot maps of EAAF. Though stacking of binary SDMs has received some criticism, most analysis has shown that stacked binary SDMs using individual species-specific thresholds based on probability of occurrence can yield accurate results [56]. Though various approaches have explored the value of different thresholds for delineating minimum suitability [57], stacking of SDMs to calculate species richness can be accurately achieved when binary maps are accurate. Over-prediction is more often associated with climate-based stacked SDMs, but rarely with habitat-based SDMs [53]. In addition, we used 10th Percentile threshold which has a tendency to either predict diversity accurately or to be conservative and under-predict diversity [55], thus we minimized the chances of inflating estimates of richness. For further information on model parameters, see S1 Appendix.

Tabulate intersection was used to calculate size of areas of different levels of biodiversity within different administrative boundaries (World Administrative Area database, www.gadm.org). Areas of migration variability (areas with the biggest differences between breeding and non-breeding phases) were identified by computing the standard deviation between breeding and sedentary non-breeding hotspot maps using the cell statistics tool. In order to identify the model variables that best explained the seasonal variability, we grouped and compared the mean values of the contribution of each variables from all the models into four different migratory phases (non-breeding, breeding, and two migratory phases).

To understand the dependence of waders on certain sites during different migration periods, both corrected temporal weighted endemism (corrected temporal WE) and temporal weighted endemism (temporal WE) were calculated using the SDM toolbox extension of ArcGIS. Though endemism is generally used to refer to species limited to a certain geographic area, here we used it to describe species which were limited to a certain area along the EAAF for a period of time, and which was presumably linked to the availability of specific resources in that location. Thus, we use the term “temporal endemism” to refer to the limited distribution of species during certain migratory phases even if they might be common across the flyway or globally indicating the importance of these areas of the EAAF for that particular species and potentially acting as a bottleneck, as these regions may have limited capacity. The temporal WE emphasizes areas with a high proportion of waders that only occur in a restricted area. The corrected temporal WE emphasizes areas which have a high proportion of waders with restricted ranges, but are not necessarily species rich.

Lastly, we compared the PA coverage to our SDM biodiversity hotspot map by tabulating areas of MaxEnt output under Protected Area status (The World Database on Protected Areas, http://www.protectedplanet.net/, release on October 2017). Based on information within the Protected Planet website (https://www.protectedplanet.net/c/calculating-protected-area-coverage), proportion of wader distributions and wader biodiversity hotspots within PAs were calculated in order to identify PA coverage throughout an annual migration cycle and during each migration period.

### 2.2. BirdLife range maps

As a comparison to our SDM modelling approach, we used existing expert-derived range maps from BirdLife database [21] to generate comparable biodiversity hotspot maps and investigate the difference between the expert maps and our SDM-derived maps. The BirdLife maps on species distributions were compiled from a variety of sources, including published and unpublished data, museum specimen localities, BirdLife’s Point Locality Database and in the Global Biodiversity Information Facility (GBIF: http://www.gbif.org) [21].

Areas of difference between biodiversity hotspot maps generated by SDMs or derived from the BirdLife International database were identified by subtracting the hotspot map from MaxEnt outputs with that from BirdLife range maps.

### 2.3. Testing accuracy

To examine the accuracy of the maps based on SDM models relative to the BirdLife maps we downloaded the GBif data for each species for 2016 (7788 points, as these data were not used for model construction) and calculated the percentage of these distribution points that fell within the ranges suggested by MaxEnt models and BirdLife range maps, for each species with at least 5 points to test models. We also calculated and compared the percentages of the EAAF mapped as suitable under each of the two approaches as, through chance alone, larger mapped ranges would be more likely to include a greater percentage of distribution points.

## 3. Results

### 3.1. Wader biodiversity patterns

**Fig 1:**
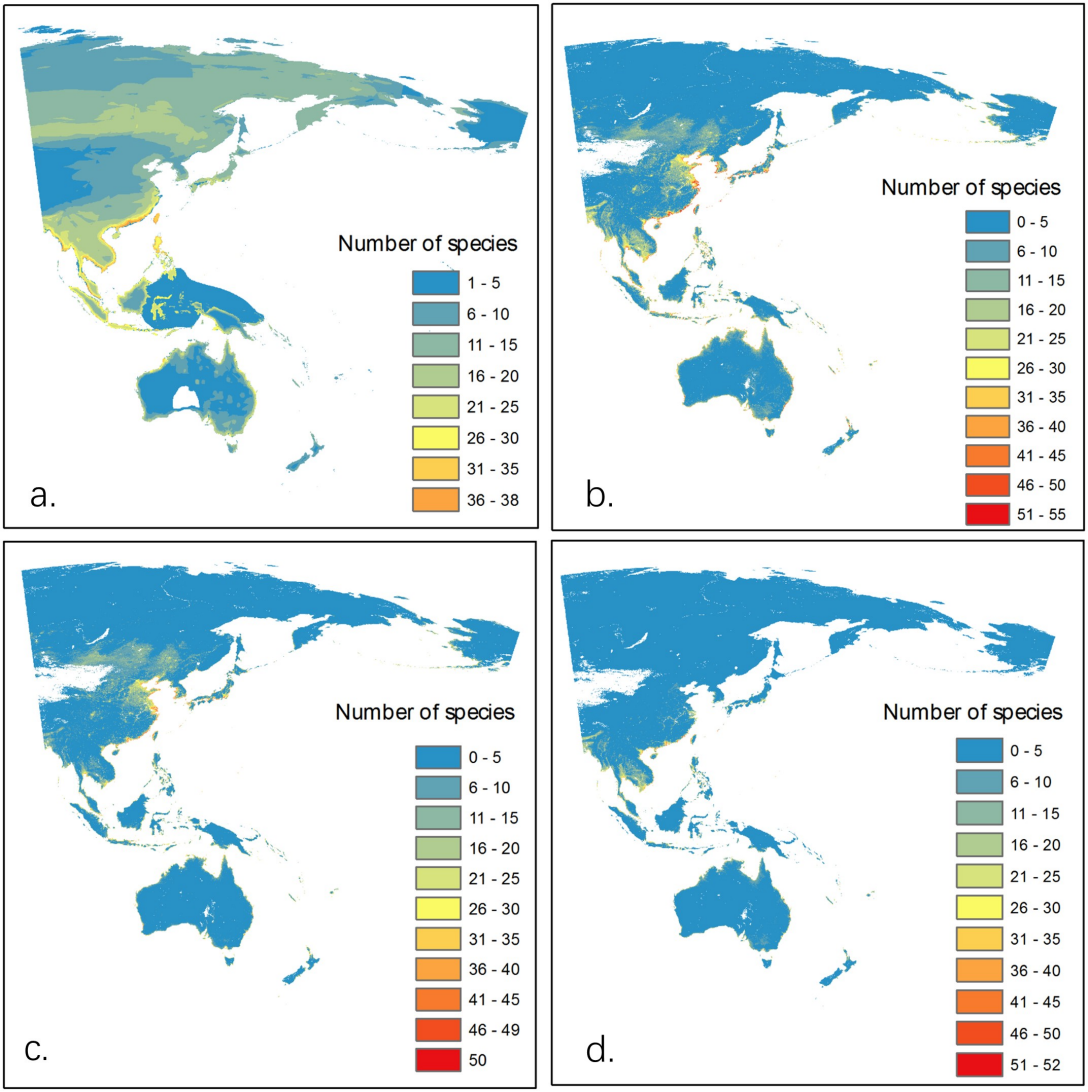
Biodiversity patterns on the EAAF. a). Wader biodiversity hotspot map generated by aggregating maps downloaded from BirdLife database. (b). Overall mean wader species richness as estimated by aggregating SDM results of 57 species. (c). Wader species richness during breeding and (d) non-breeding periods as estimated by aggregating SDM results of 57 species. Numbers indicate species richness.

**Fig 2:**
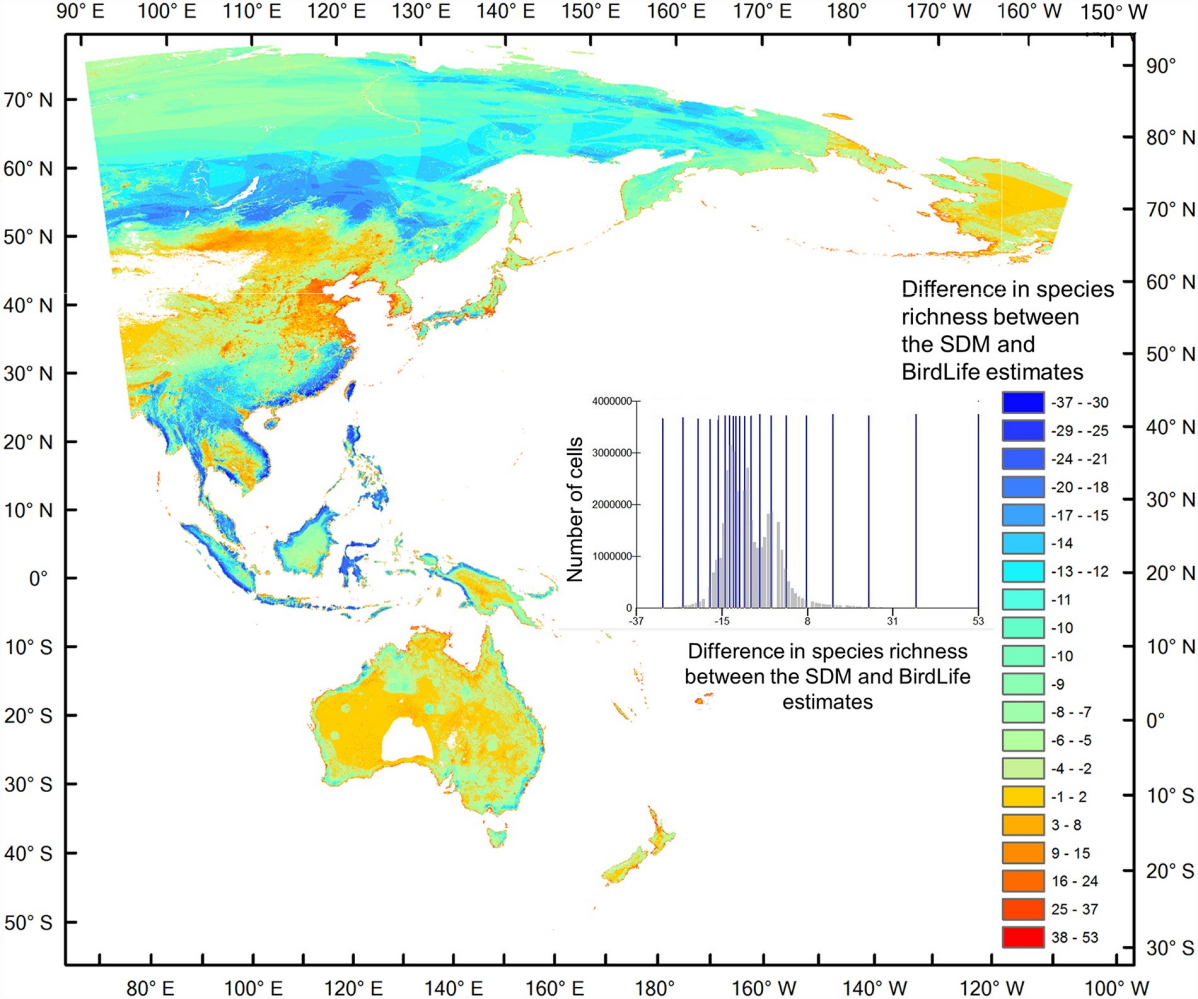
The difference between the SDM and BirdLife wader species richness estimates, calculated by using our SDM diversity map substracting the BirdLife diversity map. Positive values (Reds) indicate that SDM estimated greater species richness than BirdLife data in those regions, whereas negative values (Blues) indicate SDM estimated lower species richness than BirdLife data. Nested graph shows geometrical intervals of cell values, which was used to visualise the data. X axis shows the number of species difference. Y axis shows the number of cells.

The overall maximum wader species richness estimated by SDMs was higher than that from BirdLife range maps (Tables 1 and 2). Wader species richness from both approaches generally increased towards the equator, and was higher along coastlines. However, wader diversity on the BirdLife range maps indicated a much larger area as suitable for the majority of species while the SDM-derived maps were by and large much more restricted to a coastal belt (Table 3). Besides the difference in overall species richness, there were also considerable differences in geographical hotspots (Figs 1 and 2). The BirdLife range maps showed slightly higher species richness in much of the northern Asian region (north of 50’ N), while the SDM maps showed that the coastal wader biodiversity between 50’N and 30’N could have been greatly under-estimated in the expert assessments by up to 53 of the 57 species, particularly in the Yellow Sea ecoregion (Fig 2). Wader diversity in Australia may also have been under-estimated by the BirdLife range maps (Table 3). Other areas with under-estimated diversity included narrow coastal strips of Alaska, which were estimated to host as many as 47 of the 57 species within a single site using the SDM approach, but only 38 species in the BirdLife range maps (Fig 2). While not representing the greatest share of high species richness, large parts of Mongolia and the Russian Far-east also had a large area where wader biodiversity appears to have been under-estimated by former expert assessments (Fig 2, Table 3).

**Table 1:** The ten countries/regions with the highest wader biodiversity (ranked by maximum value of species richness), as estimated from maps drawn by BirdLife International experts and aggregated for the purposes of this study.

ID	Name	Area (km^2^)*	Min†	Max†	Range†	STD†	Median†	Mean†
1	China	10,008,651	1	38	37	5.98	9	10.24
2	Malaysia	323,414	1	38	37	7.14	22	21.11
3	Taiwan	41,499	1	38	37	3.51	34	33.95
4	Myanmar	750,043	1	36	35	5.45	19	20.35
5	Vietnam	353,279	1	35	34	6.57	22	23.23
6	Bangladesh	85,518	1	34	33	6.24	22	22.32
7	Cambodia	188,398	1	34	33	5.67	17	19.04
8	Philippines	304,795	1	34	33	4.51	27	26.81
9	Thailand	545,008	1	34	33	4.98	19	20.69
10	Brunei	5,703	1	32	31	3.85	22	22.90

*Note: variation of the same geographical region is due to the lack of data in either BirdLife maps or MaxEnt model outputs.

^†^: Min: minimum number species occur in a pixel in the named area; Max: Maximum number of species occur in a pixel in the named area; Range: the range of species richness occur in a pixel in the named area; STD: Standard Deviation of the species richness occur in a pixel in the named area; Median: The median of species occur in a pixel in the named area; Mean: the mean number species occur in a pixel in the named area.

**Table 2:** The ten countries/regions with the highest wader biodiversity (ranked by maximum value of species richness), as estimated by SDM model outputs.

ID	Name	Area (km^2^)*	Min	Max	Range	STD	Median	Mean
1	China	8,622,962	0	55	55	8.07	4	7.05
2	Australia	7,381,765	0	54	54	6.19	3	5.15
3	Vietnam	348,274	0	53	53	12.73	7	13.18
4	New Zealand	282,782	0	53	53	9.44	5	8.81
5	Japan	505,494	0	53	53	13.48	9	14.78
6	Taiwan	40,281	1	53	52	16.81	17	21.93
7	South Korea	131,013	0	52	52	14.44	14	18.71
8	Indonesia	1,779,093	0	52	52	9.45	5	9.02
9	East Timor	13,869	2	51	49	9.99	13	15.99
10	Thailand	540,025	0	50	50	9.32	13	13.83

*Note: variation within the same geographical region is due to the lack of data in either BirdLife maps or SDM model outputs.

**Table 3:** The ten countries/regions with the highest percentages of the total underestimated wader biodiversity areas estimated by comparing SDM generated hotspot maps and BirdLife range maps. These figures denote the disparity between the richness estimates for the two approaches. Minimum, maximum, range and standard deviation (STD) values are of the difference between two estimate approaches, hence a positive value indicates SDMs estimates greater species richness in the cells compared to BirdLife estimates while a negative value indicates a lower species richness from the SDM biodiversity map compared to BirdLife estimates.

Name	Area (km^2^)	Percentage	Min	Max	Range	STD
China	1,918,834	30.5%	-37	53	90	8.35
Australia	1,790,515	28.5%	-27	53	80	4.77
Mongolia	678,483	10.8%	-19	19	38	7.03
Alaska	502,735	8.0%	-13	41	54	5.29
Russia	236,427	3.8%	-21	47	68	3.52
Indonesia	203,714	3.2%	-30	50	80	9.20
Japan	169,713	2.7%	-20	51	71	13.00
Thailand	101,832	1.6%	-32	48	80	8.53
New Zealand	99,163	1.6%	-11	46	57	8.37
Others	589,269	9.4%	-35	51	86	10.06

**Fig 3:**
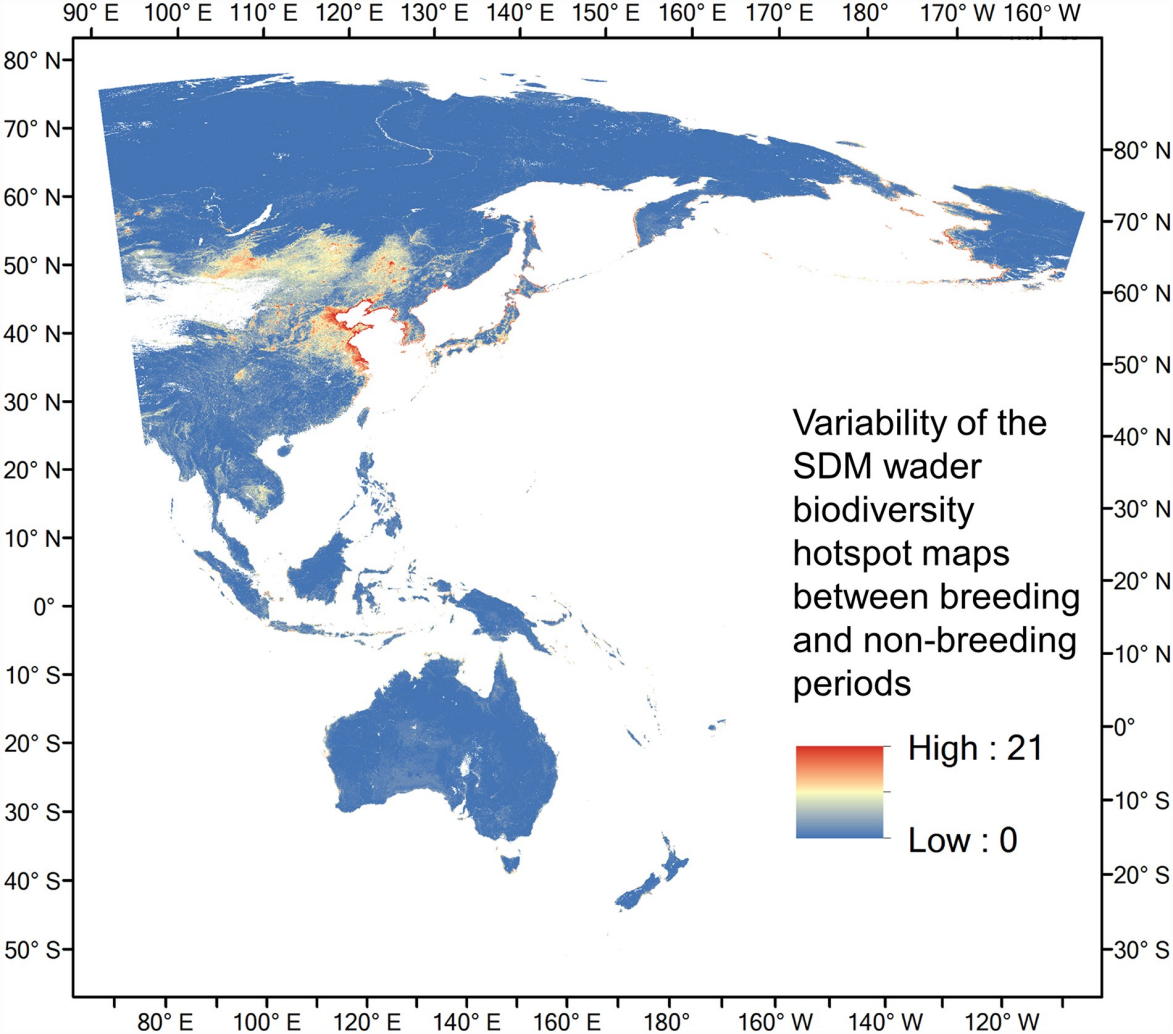
Variability (calculated using standard deviation) of the SDM wader biodiversity hotspot maps between breeding and non-breeding periods, denoting areas which show the greatest changes in species richness across the year, and therefore showing areas of seasonal importance.

East Asia, particularly around the Yellow Sea region, had large differences in species richness between breeding and non-breeding stages (Fig 3), reflecting the migration-induced variability in species richness and the relative importance of this region during the breeding phase for as many as 21 wader species. The estimated species ranges of the bar-tailed godwit (*Limosa lapponica*) is used to illustrate the discrepancy between results from the two different approaches, particularly in the Yellow Sea region (S2 Fig).

**Fig 4:**
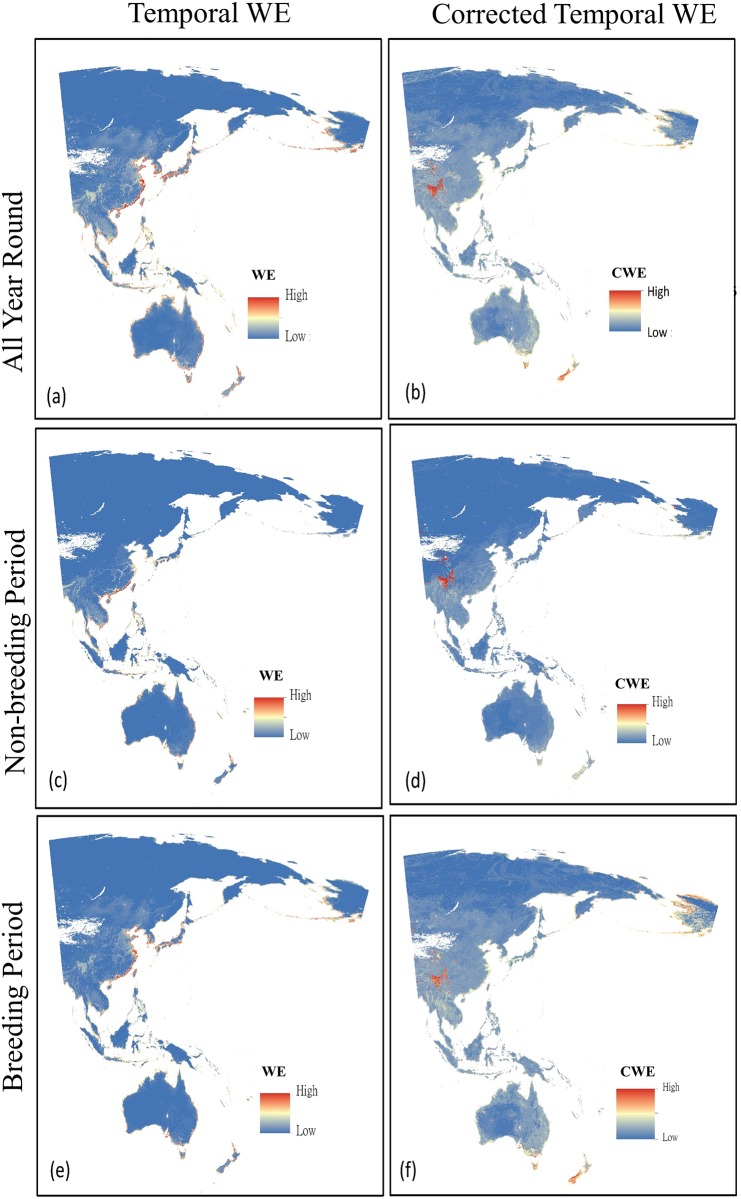
Temporal weighted endemism (WE) and corrected temporal weighted endemism (CWE) of waders of the EAAF. (a) year-round temporal WE; (b) year-round Corrected WE, (c) Temporal WE during non-breeding periods; (d) Corrected temporal WE of breeding periods; (e) Temporal WE during breeding periods, and (f) Corrected temporal WE during non-breeding periods. Within each period these denote aggregations of temporally range restricted species, therefore showing key sites for conservation of species with specialist or narrow requirements during certain parts of the year.

In terms of temporal endemism (Fig 4), coastal regions in eastern and southern China had high temporal WE values and high diversity of waders. Conversely, Alaska, New Zealand and the transitional zones between Tibetan plateaus to lowland areas in South Western China had high Corrected temporal WE values.

### 3.2. Environmental drivers of wader species distributions

**Fig 5:**
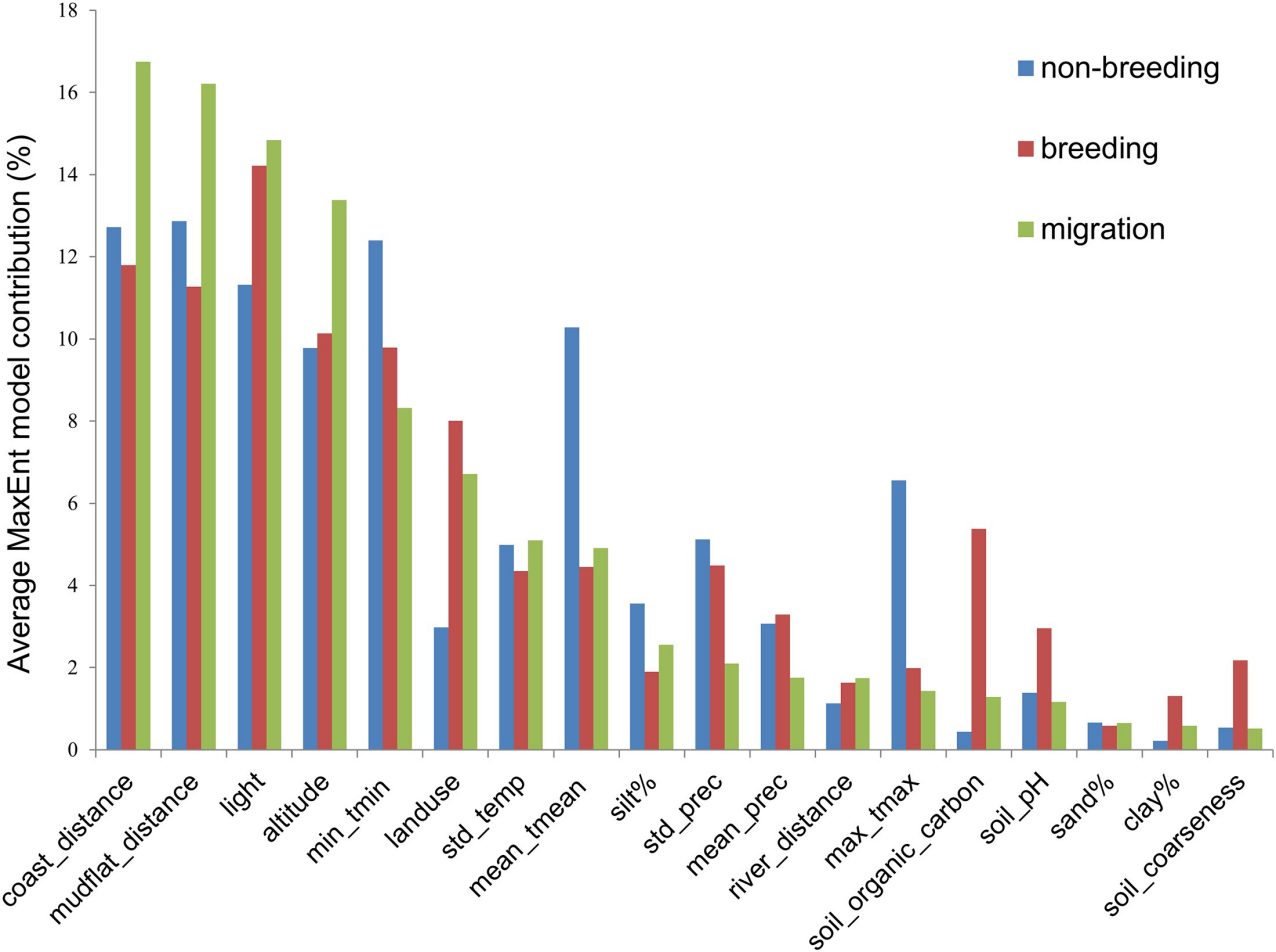
Model variables and their relative contribution to model results during different migratory phases. Altitude: altitude; Clay %: clay percentage; coast_dist: distance to coast; landuse: landuse; light: night-time light; Max tmax: maximum of monthly maximum temperature; Mean tmean: mean of monthly mean temperature; Min tmin: minimum of monthly minimum temperature; mudflat flat_dist: distance to intertidal flat; river_dist: distance to river; sand%: sand percentage; silt%: silt percentage; soil_c: soil coarseness; Soil_organic_c: soil organic matter; STD precipitation: standard deviation of precipitation; STD temperature: standard deviation of monthly temperature.

Overall, the most important environmental variables influencing wader distributions over an annual migration cycle were, in decreasing order of importance, distance to coast, distance to intertidal flats, night-time light, and altitude, each of which contributed on average at least 10% towards determining species occurrence across all migratory phases (Fig 5). Land use, lowest of monthly minimum temperature, and the monthly mean temperature were also important throughout the year with an average of around 5% contribution each.

A number of environmental variables were more influential during specific migratory phases. While altitude, distance to coast and distance to intertidal-flats were important factors throughout the year, their contributions to wader distributions were more significant during migration than during the non-breeding or breeding phases (Fig 5). In contrast, the distributions of waders during the non-breeding phase was more sensitive to temperature variables and precipitation variability. Soil parameters and precipitation variation had a much greater influence on wader distributions during the breeding period relative to other migratory phases (Fig 5).

### 3.3. Protected area coverage

**Fig 6:**
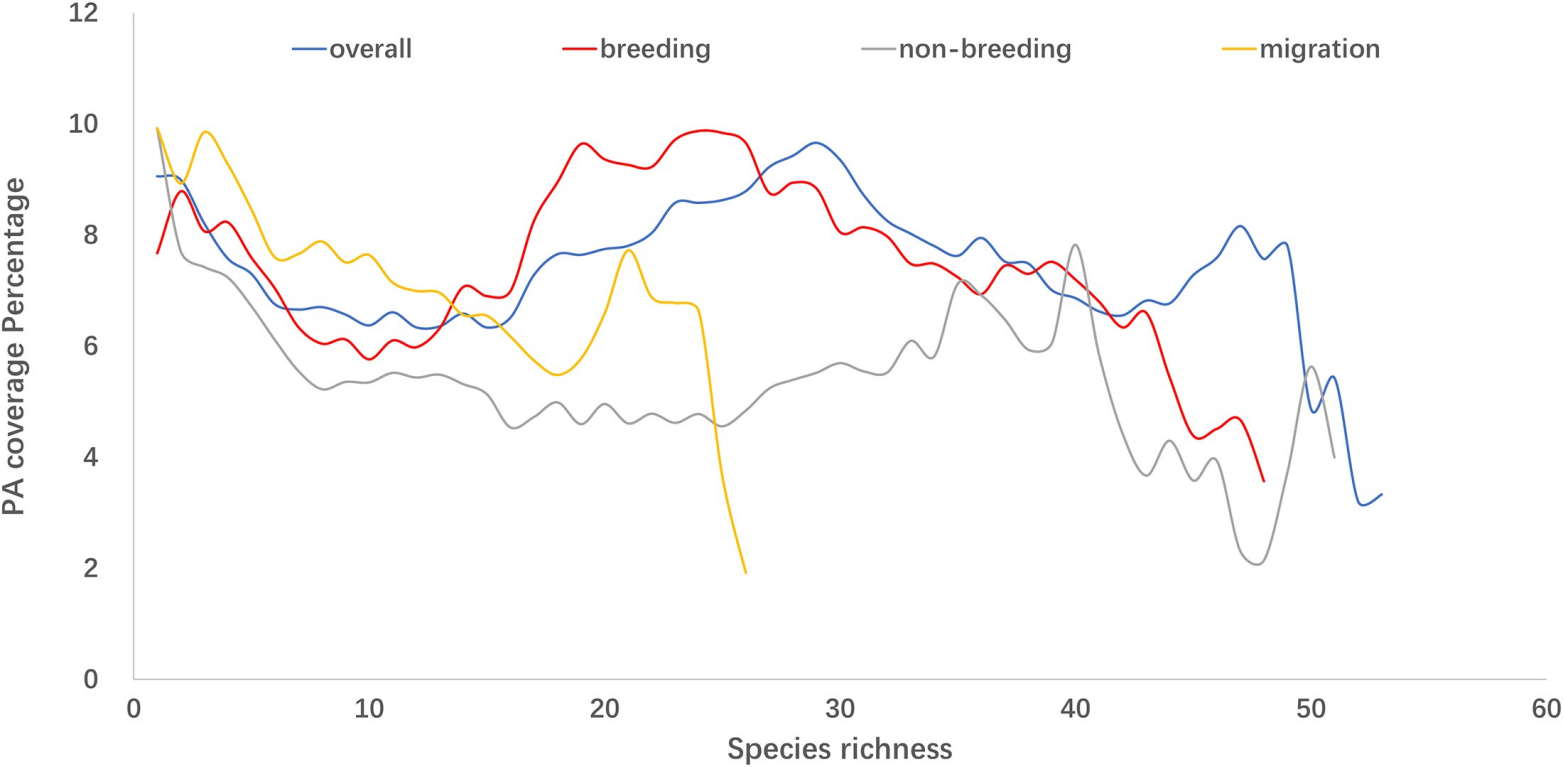
Protected Area (PA) coverage of wader ranges at different species richness and during different migratory phases.

**Fig 7:**
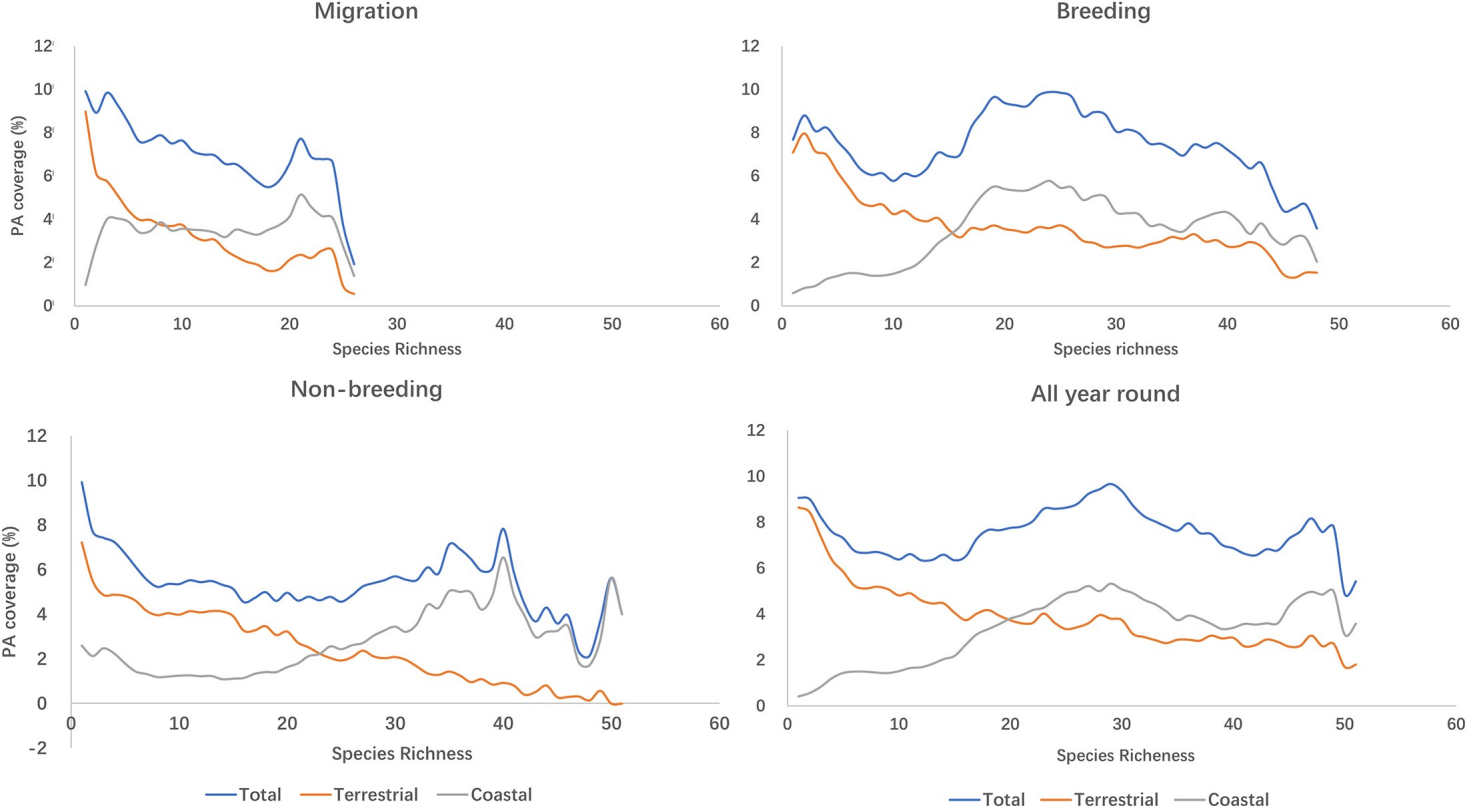
Protected Area (PA) coverage by types of wader ranges at different species richness level during different migratory phases.

PA coverage of species-rich areas for waders was generally low. PA coverage generally decreased slowly as the level of diversity increased. On average, only 8% of the areas with wader distribution is within PA boundary (7% terrestrial PA and 1% coastal PA) (Figs 6 and 7). Areas with high species richness (top 20%, or over 45 of the 57 species) had a 6% PA coverage (Fig 6).

**Fig 8:**
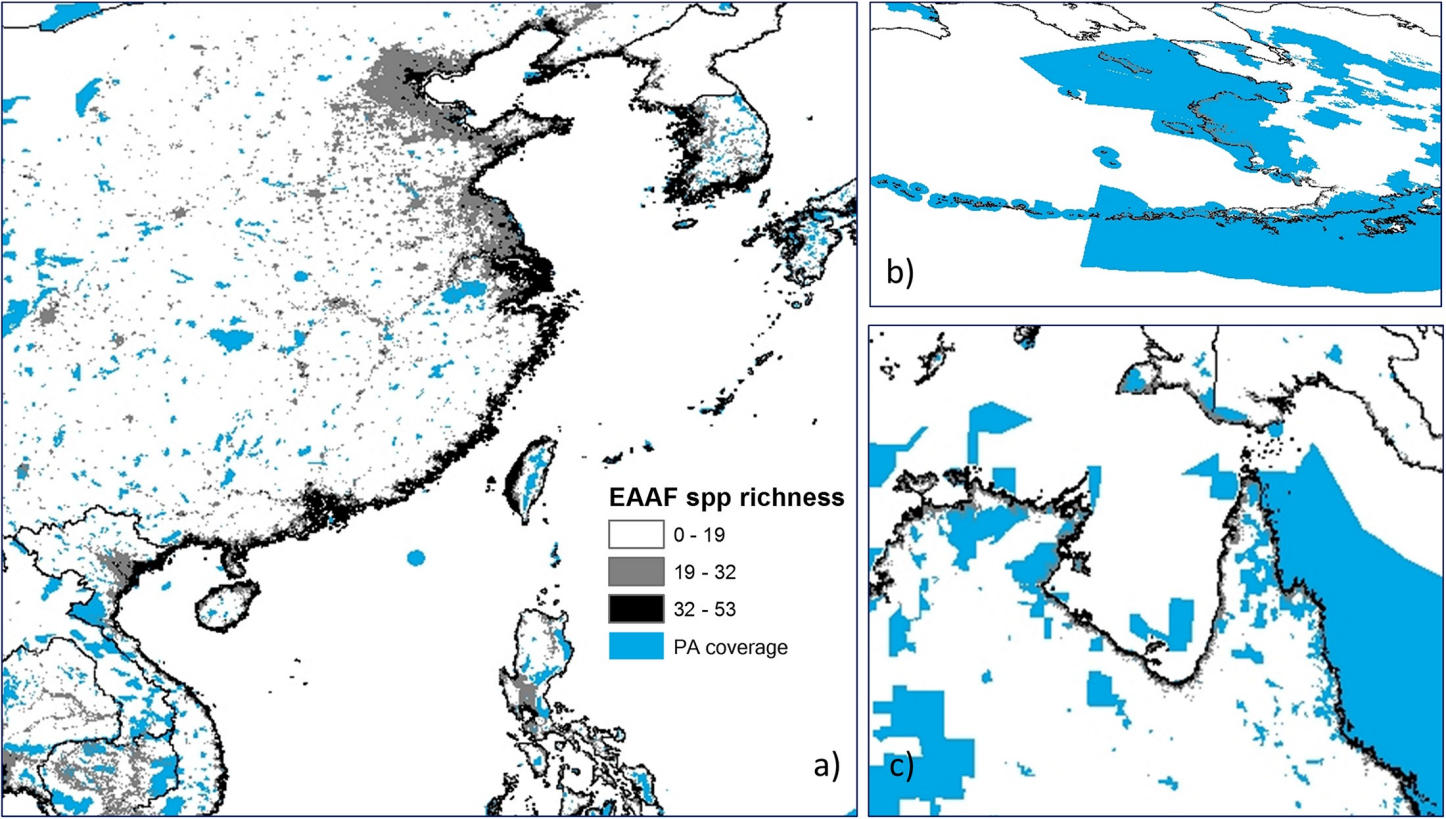
Species richness within protected areas (PA). a) East Asia, Yellow Sea and part of south East Asia region; b) Alaska c) northern Australia and part of pacific.

Furthermore, most of the areas along the EAAF that had PA status were situated inland, leaving the majority of coastal areas unprotected. Unfortunately, these were the sites that typically had the highest wader diversity, most particularly, throughout Eastern China, Yellow Sea region and Southeast Asia (Fig 8).

### 3.4. Testing accuracy

**Fig 9:**
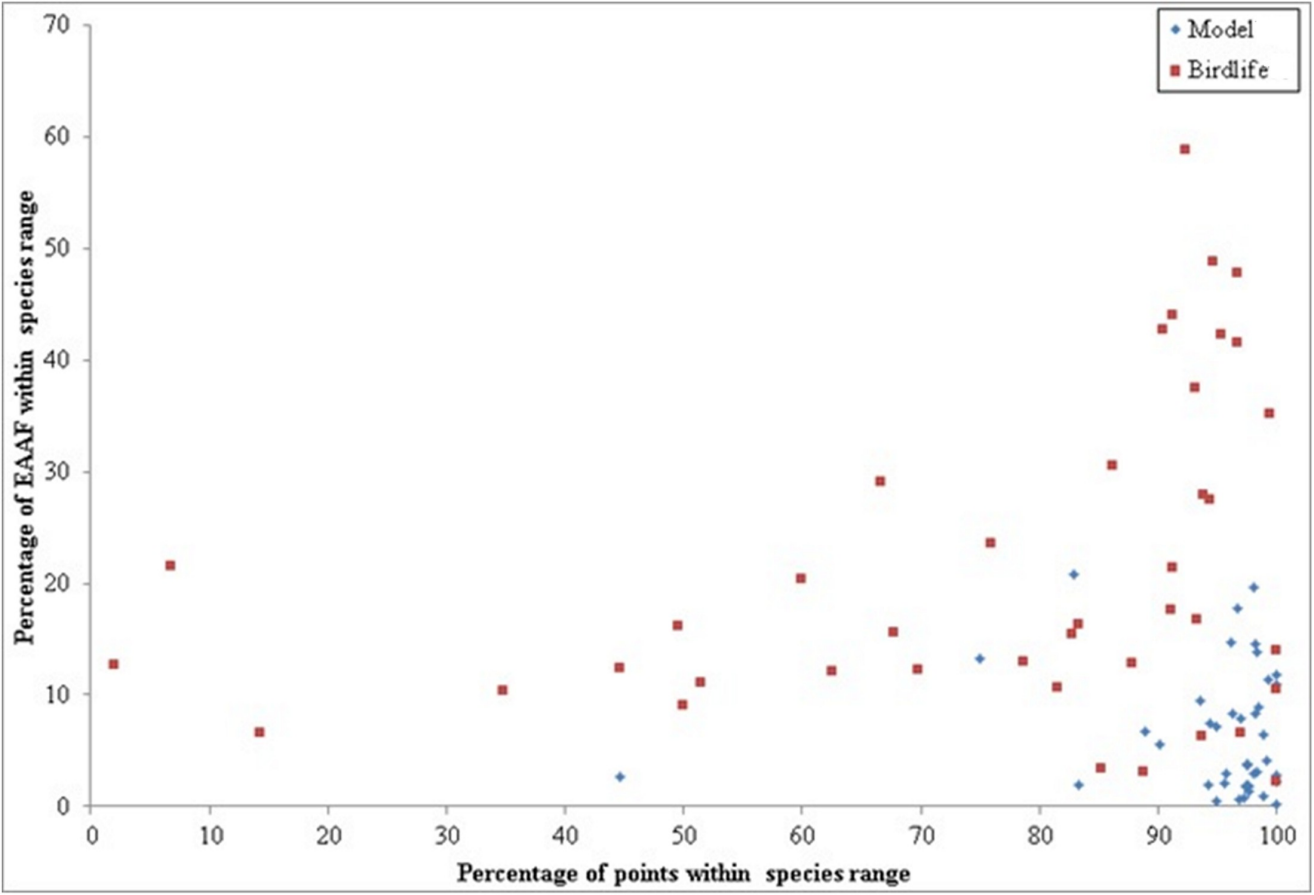
Accuracy compared to area coverage. MaxEnt modelled ranges are more restricted (6.6% of the EAAF) but with high accuracy (average 95%), whereas BirdLife designates a much larger area as suitable (up to 60% of the region and with a mean of 21.1%) but has a much lower accuracy (76.5% on average, but as low as 2%).

Based on our models, an average of 94.6% occurrence locality points fell within the ranges predicted by SDM mapping, whereas only 76.5% of points fell within the expert-derived range maps. Yet, in terms of area coverage, the expert-derived maps covered 21.2% of the EAAF, and the SDM maps only 6.6%. This means that despite the SDMs predicting smaller range areas, the predictions match better the recorded distributions for almost all species examined compared to the expert-derived maps (Fig 9). Only a single species, the grey-headed lapwing (*Vanellus cinereus*), showed an accuracy under 75% in SDM maps, whereas 17.1% of expert-derived maps had lower than 50% accuracy and 31.5% had an accuracy of under 75%. The expert-derived BirdLife maps showed higher accuracy than SDM-derived maps for only 7.3% of species maps. For those species where the expert-derived maps had higher accuracy, they predicted 12.2% of the total mapped area to be suitable whereas SDM-derived maps yielded a mean of just 2.5%, suggesting that the higher accuracy could have been due to chance alone (arising from the greater predicted range extent) and highlighting the need for more sampling. In general, SDM-derived maps had higher consistency, with a standard deviation of 9.5% in model accuracy, whereas expert-derived range maps had a standard deviation of 26%.

## 4. Discussion

In this study, we present the first flyway-scale map of wader biodiversity for the EAAF, and compared the patterns of wader diversity generated by BirdLife range maps versus those based on SDM. We highlight the fact that the wader biodiversity in many parts of the flyway has formerly been underestimated, and that there is significant spatial variability in the usage of different areas along the flyway. The results have significant implications for effective conservation of migratory waders and the protection of critical areas used during their migratory phases. Developing conservation priorities and management protocols requires up-to-date knowledge of diversity hotspots, and out-of-date or inaccurate maps may risk unwise investment in site protection at the expense of more diverse areas, and may result in the connections between critical areas of habitat being overlooked.

### 4.1. Conservation priority areas

To our knowledge, the present study provided a first quantitative hotspot map of avian biodiversity of the EAAF, using the species richness of a subset of migratory waterbirds as a proximate indicator of other waders which also utilise the EAAF. Our analysis showed that avian species richness along the EAAF might be greatly underestimated in many regions, including the Yellow Sea areas, Australia, Mongolia, Alaska and potentially some Pacific islands. Furthermore, we assayed the adequacy of protection of various biodiversity richness, and demonstrated that current PA coverage is low in the most species-rich areas (less than 10% PA coverage in general and less than 6% for the top 20% of species rich areas). The 7% terrestrial PA coverage and 1% coastal PA coverage are even more alarming, which is significantly less than the 17% terrestrial PA coverage and 10% coastal and marine PA coverage envisaged under Aichi Target 11. Though, this analysis did not include marine PA so our result of coastal PA coverage is not directly comparable. Nevertheless, these figures are sufficient to show that at least for the purpose of wader conservation, countries along the EAAF are not on track to achieve their Aichi Target 11.

Several biodiversity hotspots revealed using SDM were missing from the hotspot map we generated from the BirdLife International range maps. In particular, Yellow Sea has been repeatedly identified as a major stopover site for migratory waterbirds [14, 34, 58], but the importance of this region is currently not reflected on BirdLife maps.

Our study showed that the Yellow Sea region had higher species richness than shown in the biodiversity hotspot map generated by stacking BirdLife range maps of the same species (Fig 2). Our SDM results also showed that the region was frequently used by migratory waders (Fig 3). These trends were confirmed by existing knowledge [59] and data from more recent surveys [60, 61]. While the BirdLife Redlist assessments of individual species have also included Yellow Sea as a key stopover sites, their range maps require updating. Importantly, the Yellow Sea region was among the most poorly represented regions within the EAAF PA network (Fig 7a). Our SDM distribution maps also accord with field observations from national bird censuses and published reports that underline the exceptional biodiversity around the Yellow Sea [11, 60]. According to the China Coastal Waterbird Census, 1% of the global populations of 42 waterbird species were recorded at least once during 2005–2013 at a number of sites around the Yellow Sea [60], qualifying them for designation as Wetlands of International Importance [62].

Unfortunately, the coastal regions of Yellow Sea are experiencing unprecedented development pressure, particularly in Eastern China and South Korea [11, 58]. A recent study showed that the summer populations of three migratory wader species in Australia—the bar-tailed godwit (*Limosa lapponica)*, red knot (*Calidris canutus piersmai*) and great knot and (*C*. *tenuirostris*)—had declined as a result of habitat loss in the Yellow Sea region where these birds stopover during migration [14]. Additional evidence linking the decline of wader populations with coastal development and deteriorating water quality in the Yellow Sea has been reported for the Far Eastern curlew (*Numenius madagascariensis*) and curlew sandpiper (*Calidris ferruginea*) [34], as well as a number of other shorebirds that use this region [4]. Furthermore, when comparing shorebirds with varying degree of reliance on Yellow Sea region, Studds (2017) found that species using it as stopover site had higher rates of population decline than those using other regions as primary stopovers. These declines could result from changing tidal regimes due to costal development and land reclamation [63, 64] that have reduced foraging habitat, increasing levels of pollution in the intertidal zones of Yellow Sea [65, 66], as well as the spread of invasive intertidal plants in the region [67].

The flyway concept is an aid to conservation and management as it serves to link different geographical regions so that the complete life cycle of migratory birds can be discerned at a scale that transcends national or other administrative boundaries. We showed that Yellow Sea coastlines should be a conservation priority for the EAAF flyway, because of the high biodiversity level and the inadequate protected-area coverage of this critical area [58, 68]. However, many of the diversity hotspots we identified are within estuarine areas bordering major coastal cities (e.g. Shanghai, Tianjin and Seoul). Given the development pressure and high land prices in the coastal regions of China and South Korea, it may be difficult to increase the extent of protected areas. Pragmatic strategies for facilitating the persistence of birds in human-dominated coastal landscapes are needed [69–71], and will be useful to prevent further population declines of the wader species that rely on sites in proximity to human settlement during periods of their annual cycle. Anthropogenic coastal wetlands such as saltpans and sustainably managed aquaculture ponds could play an important role in partially mitigating the loss of natural wetlands [61, 72]. It is worth noting though that engineering intertidal habitats for species conservation purposes can be complex due to multiple factors at play (natural tidal cycles, harvesting regimes, management incentives and ecological response of target species, etc.) [72–74].

### 4.2. Environmental drivers of wader distributions

The model results confirmed that intertidal-flats are key habitats for waders, particularly during migration [75–77]. Although land use was often an important environmental factor, our model results indicated that its importance was overshadowed by other factors such as altitude, and distance to coasts or to intertidal-flats. This means that the most relevant land-cover-related factor for wader distribution was coastal mudflat. Availability of intertidal-flats was particularly critical during north- or southward migratory phases, though soil type and organic content were also important variables during breeding periods [78, 79]. We had anticipated that waders would avoid highly disturbed areas [80–82], but the lights at night (a proximate indicator of disturbance and modification) turned out only to be the third most important predictor. This lack of avoidance of disturbed areas could potentially be explained by the proximity of city locations and the remaining coastal habitats. Nonetheless, our study found that wader species richness was higher along the coast relative to inland areas, especially during migration.

Environmental variables had an unequal influence on wader distribution in different migratory phases [83, 84]. As shown in Fig 5, temperature variables, particularly the lower and upper limits, seemed to be more important during sedentary non-breeding compared to other periods, likely reflecting physiological tolerances or temperature requirements during that period, or the availability of prey during cold periods [85, 86]. Variation in precipitation was more important during breeding and non-breeding periods than during north- or southward migration when other factors (i.e. topography and feeding grounds) were important [87]. The higher importance of soil parameters and precipitation variables during breeding phase is probably a result of the need for higher volumes of nutrient rich food to feed offspring [87, 88].

### 4.3. Temporal endemism

Many wader species are known to use inland habitats during parts of their migration periods [20, 89]. In this study, the transitional zones between the Tibetan plateau and more lowland areas in southwestern China coincided largely with the mountain regions of southwestern China, a biodiversity hotspot formerly identified by Conservation International (CI), plus some adjacent small parts of the Himalayan region, which has also been listed as a biodiversity hotspot area by CI [90, 91]. This area also includes in several Endemic Bird Areas (EBA) identified by BirdLife International, such as southern Tibet and the eastern Himalayan and Yunnan mountains [21, 92]. Observational reports also support our SDM model findings of the presence of coastal waders in inland southwestern China [93].

The existence of inland temporal endemism hotspots reinforces the message that these non-coastal habitats are also important for waders during key migratory phases, particularly species capable of long-distance migration [94]. While coastal regions function as a migratory highway, inland habitats also provide stopover and non-breeding sites for migratory waders [95], although few studies have investigated the relative importance of inland habitats for these birds [95, 96]. Given the intensity of current development pressures along coastal regions [12], many wader species could lose important coastal stopover sites and may shift to exploit inland areas during migration [93]. The impacts of such shifts on post-migratory fitness are difficult to estimate but are hardly likely to be positive, and will depend on the additional distances travelled as well food availability at inland stopover sites [67].

Not all wader species can make use of inland habitats [97, 98]. The development of coastal fringes and land-use changes that impact individual inland sites increases the distances between stopovers and thus reduces individual survival probability and breeding success [99, 100]; the impact of such habitat loss would also be magnified through the need to maintain connectivity among habitats required at different stages of the annual life cycle [4, 8]. More field studies are needed to better understand the ecological significance of inland habitats for migratory waders, and to determine whether and how many shorebirds can adapt to—or be supported by—them. A combination of ringing studies and population monitoring would be needed to assess when and how inland habitats are used by different species, and could provide crucial data to inform management of wader populations as coastal regions continue to be developed.

### 4.4. Estimating species distributions and diversity

This study showcases a relatively rapid method to estimate wader biodiversity based on field observations such as eBird records and relatively user-friendly SDM techniques. Our modelling results provided new insights into where the hotspots are, how they compare to those formerly estimated using alternate methods of analysis, and how they shift across the year. It provided confirmatory evidence of the conservation importance of key stopover regions in the Yellow Sea region [59, 67], which are necessary to maintain connectivity between breeding and non-breeding grounds, and increasingly draw attention from the international conservation community [20].

Our model accuracy tests used data from Gbif database which is known to suffer from quality issues [101, 102]. Nevertheless, combining with observation records and existing literature [25, 27, 60], the larger range yet lower accuracy among birdlife range maps suggested a tendency of over prediction compared to SDM outputs [103]. SDM predictions were supported in more frequently surveyed areas such as the East Asian region (i.e. Japan, Hong Kong and some parts of Eastern China [60, 104], while showing discrepancies with hotspot maps based on BirdLife range maps in less surveyed areas such as central Australia, Mongolia and the Russian Far East.

While large datasets can have shortcomings arising from geographical bias [105], the ready availability of data from citizen scientists through eBird and i-naturalist has enabled the development of extensive datasets with greater geographic extent and higher sample sizes than could be gathered by scientists alone [106]. In comparing extent of occurrence (EOO) and extent of suitable habitats (ESH) using BirdLife range maps and model estimates of 157 avian species in Africa, Beresford et al. [107] found that ESH of a species typically comprised of only 28% of its EOO. They also found high levels of commission and omission errors after using an independent field data set to verify EOO estimate [107].

Our SDM outputs are not intended to contest the usefulness and importance of BirdLife expert-derived maps. Instead, they highlight the need for further study in some key areas, to improve and update our knowledge of the habitat use of migratory birds in these areas. For example, although recent studies and observation records indicate bar-tailed godwit dependence on Yellow Sea coastal zones [34, 60], the species range map is yet to be updated in the BirdLife Database (S2 Fig). The impacts of outdated range maps should not be underestimated as the IUCN Redlist is one of the most commonly referenced and used conservation tools [108] and has made its way into both academic research [22, 24] and real life applications in public [109] and private sectors [110]. One of the benefits of using SDM of multiple species as a methodology for identifying hotspots is that it facilitates the evaluation of potential habitat in regions that cannot be easily surveyed, but which show similar conditions to areas that can be surveyed. This is important given the urgent need to advance our knowledge of the overall biodiversity of the EAAF in the context of escalating development pressures in coastal areas, as well as likely changes in land-use at more inland sites of potential importance for waders.

## 5. Limitations

This study presented a novel approach to estimate migratory species distribution, but the approach also has its limitations. Compared to well-designed species population surveys, sampling efforts based on large scale citizen science data is uneven. For example, although sampling included most of climate-space occupied by 57 study species, more northern latitudes (and especially the arctic) are likely to have been under-sampled due to remoteness, and their importance may consequently be under-estimated [111]. While our SDM-derived maps can support site prioritisation and flyway management in tropical-temperate zones, we caution that they may underestimate the importance of boreal and arctic regions which are known breeding grounds for many waders but are likely to be under-represented in our approach due to the lack of sampling in these regions. Our approach also focus on species richness which is only one facet of biodiversity. In designated important wetlands, decision makers are also advice to include other consideration such as total abundance and the presence of endangered species [20].

## 6. Conclusions

We demonstrate that using predictive analyses (SDM) to examine the changing relationship between migratory waders and their environment throughout the year can provide key insights and therefore assist in making effective conservation decisions for the management of one of the world’s largest and most important flyways. Our results clearly demonstrate that species richness in many parts of the EAAF has likely been underestimated, and that many biodiversity-rich sites occupy small areas and receive little or no protection. Current PA coverage is insufficient to protect the biodiversity hotspots along the EAAF, and thus it should be increased. How can this be achieved when key wader habitats intersect with locations adjacent to coastal cities where dense human populations and high land values result in intense pressure to develop or ‘reclaim’ intertidal areas? Successful conservation of waders along the EAAF must move away from the traditional conservation paradigm of totally separating areas used by humans and biodiversity. More thoughts and investments should be given to pragmatic strategies that can allow co-existence of city, ports and wader species, such as estuaries adjacent to large cities around the Yellow Sea region. Implementation of legislation to protect sensitive and important areas from disturbance, including disposal and leakage of chemicals and pollutants which may degrade important wetland habitats, will be essential, combined with constraints on excessive development of locations close to biodiversity hotspots. Improved understanding of the magnitude of richness and spatial distribution of critical areas along the EAAF and how they vary over space and time, based in part on the findings provided by our modelling approach, will provide a sound basis for the formulation and delivery of conservation and plans for the EAAF and its rich avifauna.

## Supporting information

S1 Appendix Using MaxEnt. (DOCX)

S1 Table List of environmental variables considered and sources, or methodologies. (DOCX)

S2 Table List of migration species and corresponding phases. (DOCX)

S3 Table Total Area by species as estimated Birdlife Range maps and by MaxEnt modelled output, and the difference using Birdlife range maps to subtract MaxEnt outputs (unit: km^2^). (DOCX)

S1 Fig Movement of great knot (*Calidris tenuirostris*) over an annual migration cycle. Top: Non-breeding period—Oct, Nov, Dec, Jan, Feb, March; Bottom left: Northward migration—April, May; Bottom middle: Breeding period—June, July; Bottom right: southward migration—August, September.
(TIF)

S2 Fig Part of the distribution of *Limosa lapponica* (Bar-tailed Godwit) as estimated by MaxEnt output and BirdLife range map. (TIF)
