# Correction: Mapping wader biodiversity along the East Asian—Australasian flyway

**DOI:** 10.1371/journal.pone.0215877

**Published:** 2019-04-18

**Authors:** Jia Li, Alice C. Hughes, David Dudgeon

The Data Availability statement for this paper is incorrect. The correct statement is: Model data is available from the GBIF database at https://doi.org/10.15468/dl.cp0csd. All other relevant data are within the manuscript and its Supporting Information files.

In the 2.3 Testing accuracy subsection of the Materials and Methods, a reference is omitted from the first sentence. The correct sentence is: To examine the accuracy of the maps based on SDM models relative to the BirdLife maps we downloaded the GBIF data (GBIF.org, accessed on 07 April 2016) for each species for 2016 (7788 points, as these data were not used for model construction) and calculated the percentage of these distribution points that fell within the ranges suggested by MaxEnt models and BirdLife range maps, for each species with at least 5 points to test models.

The reference is: GBIF.org (07 April 2016) GBIF Occurrence Download https://doi.org/10.15468/dl.cp0csd.

## References

[1] Li J, Hughes AC, Dudgeon D (2019) Mapping wader biodiversity along the East Asian—Australasian flyway. PLoS ONE 14(1): e0210552 10.1371/journal.pone.0210552 30682055 PMC6347144

