# Supplementary material for: Mapping wader biodiversity along the East Asian—Australasian flyway
Source: PLoS One. 2019 Jan 25;14(1):e0210552. doi: 10.1371/journal.pone.0210552 (PMC6347144; doi:10.1371/journal.pone.0210552)
Supplement: S1 Appendix — (DOCX) [file pone.0210552.s001.docx]

S1 Appendix: Using Maxent

In this study we use MaxEnt modelling. The modelling approach has its limitation and critiques but It is particularly attractive for conservation practitioners because of its user-friendliness. The two main types of data input it requires are recorded point distributions and environmental “layers”, both of which are now increasingly accessible to ecologists and conservationists, in part due to the increasing availability of remote sensing data [[1](#_ENREF_1)] and the sharing of bird observation records from birdwatchers via various digital platforms.

Analyses have shown MaxEnt to be conservative and one of the most accurate methods to map species distributions [[1](#_ENREF_1)]. In our case was tested projected biodiversity patterns using the published literature to validate the locations of hotspots, and especially overlooked hotspots (not included in birdlife maps) [[2-4](#_ENREF_2)] to ensure these were not the result of over-prediction in certain areas and that they accurately projected recorded patterns of diversity.

Given the scale and geographically scope of EAAF, for the purpose of this study determining high concentration of migratory shorebirds at the flyway scale), we believe MaxEnt is the appropriate tool and the results showed a great potential for this study at the flyway scale.

Reference:

1. Merow C, Smith MJ, Silander JA. A practical guide to MaxEnt for modeling species’ distributions: what it does, and why inputs and settings matter. Ecography. 2013;36(10):1058-69. doi: 10.1111/j.1600-0587.2013.07872.x.

2. Aharon-Rotman Y, Bauer S, Klaassen M. A chain is as strong as its weakest link: assessing the consequences of habitat loss and degradation in a long-distance migratory shorebird. Emu. 2016;116(2):199-207. doi: https://doi.org/10.1071/MU15029.

3. Choi Y, Lee WS, Lee C-K, Dattilo J. Valuation of Mudflats in Nature-Based Tourism: Inclusion of Perceived Value of Festival Experiences. Tourism Economics. 2015;21(4):833-51. doi: 10.5367/te.2014.0370.

4. Hua N, Tan KUN, Chen Y, Ma Z. Key research issues concerning the conservation of migratory shorebirds in the Yellow Sea region. Bird Conservation International. 2015;25(1):38-52. doi: 10.1017/S0959270914000380.
