# Supplementary material for: Mapping wader biodiversity along the East Asian—Australasian flyway
Source: PLoS One. 2019 Jan 25;14(1):e0210552. doi: 10.1371/journal.pone.0210552 (PMC6347144; doi:10.1371/journal.pone.0210552)
Supplement: S1 Table — (DOCX) [file pone.0210552.s002.docx]

S1 Table. List of environmental variables considered and sources, or methodologies

| Environmental variables | Sources of data |
| --- | --- |
| Altitude, precipitation and temperature | Altitude data was downloaded from worldclim.org. Precipitation and temperature of specific migration periods are produced using mean, standard deviation, maximum and minimum statistics of the climatic factors for each month of corresponding time period. For each migration period, a set of 6 climatic factors are produced (mean temperature, mean precipitation, minimum temperature, maximum temperature and the standard deviation of precipitation, and the standard deviation of temperature). See Table S2 for a full list of migration species and the corresponding migration periods over which these cell statistics were conducted. |
| GlobCover 2009 (Global Land Cover Map) | European Space Agency ( http://due.esrin.esa.int/page_globcover.php) |
| Distance to intertidal-flat | As there is no existing flyway scale mudflat map, we modelled a map of suitable intertidal-flat across the region (see below) and then calculate the distance to nearest suitable intertidal habitats using the path distance tool. Though maps of mudflats for at least some parts of the region have been created using various methods (i.e. see [[5](#_ENREF_5)].[[6](#_ENREF_6)]) these are only currently available for part of the region; thus a new map of mudflats using consistent methodology across the region needed to be developed.  First, a 30m resolution Digital Elevation Model was downloaded from Shuttle Radar Topography Mission (SRTM) database (https://lta.cr.usgs.gov/SRTM1Arc) and potential areas of intertidal-flat were mapped out by buffering coastline of the study area and extracting any coastal areas with low altitude (<150 m) and low slope (below 20 degrees-calculated using the slope tool), this should be indicative of intertidal flat formation both above and below mean sea-level by accounting for the conditions likely to result in mudflat formation.  Additionally, we used the eBird observation records to inform the suitable range of soil conditions for waders, and extracted these suitable ranges of soil characteristics (silt, clay, and sand, soil coarseness, soil organic matter and soil pH: downloaded from ISRIC 1KM global soil database) using extract multiple values to points. Once wading birds requirements had been collated (using the extract multiple values to points tool), each soil variable was reclassified to give a zero for all areas which fell below the maximum bounds of suitability of those experienced by the species, and no data where the conditions were not met. The intersect tool was then used to combine these soil requirement masks, and find areas which satisfied all conditions for each of the five soil variables. Once the soil requirement layer had been developed, this was intersected with the low slope angle areas within 10km of the coastline to create a map of all suitable intertidal flat habitats. Satellite imagary within ArcMap 10.3 (Nature Vue) was used to verify the accuracy of the intertidal flat map in points across the region and ensure we were accurately mapping the locations of intertidal flats suitable for waders.  Using path distance tool in ArcGIS, the distance to mudflat variable was calculated for the purpose of this study. |
| Distance to coast | Distance to coast was calculated using the path distance tool from a coastline shapefile (http://www.naturalearthdata.com/downloads/10m-physical-vectors/10m-coastline/ ) |
| Distance to river | A map of rivers was obtained from the hydroshed database, based on which the distance to river is calculated using path distance tool. (http://www.hydrosheds.org/page/hydrobasins) |
| Soil parameters | silt, clay, sand, soil coarseness and soil pH, soil organic matter, (ISRIC 1KM global soil database) |
| Light of Night data | Downloaded from lights at night database (<http://ngdc.noaa.gov/eog/> ) |
