# Supplementary material for: Mapping wader biodiversity along the East Asian—Australasian flyway
Source: PLoS One. 2019 Jan 25;14(1):e0210552. doi: 10.1371/journal.pone.0210552 (PMC6347144; doi:10.1371/journal.pone.0210552)
Supplement: S2 Table — (DOCX) [file pone.0210552.s003.docx]

S2 Table. List of migration species and corresponding phases

| Latin names | non-breeding | M phase2 | breeding | M phase4 |
| --- | --- | --- | --- | --- |
| *Actitis hypoleucos* | 11_1 | 2_4 | 5_7 | 8_10 |
| *Arenaria interpres* | 11_1 | 2_4 | 5_7 | 8_10 |
| *Calidris acuminata* | 11_3 | 4_7 | 8_10 |  |
| *Calidris alba* | 12_3 |  | 4_9 | 10_11 |
| *Calidris alpina* | 11_3 | 4_5 | 6_8 | 9_10 |
| *Calidris canutus* | 9_3 | 4_5 | 6_8 |  |
| *Calidris falcinellus* | 10_2 | 3_5 | 6_7 | 8_9 |
| *Calidris ferruginea* | 12_2 | 3_5 | 6_8 | 9_11 |
| *Calidris ptilocnemis* | 1_12 |  |  |  |
| *Calidris ruficollis* | 10_3 |  | 4_9 |  |
| *Calidris subminuta* | 10_3 | 4_5 | 6_6 | 7_9 |
| *Calidris temminckii* | 11_3 | 4_5 | 5_8 | 9_10 |
| *Calidris tenuirostris* | 10_3 | 4_5 | 6_9 |  |
| *Charadrius alexandrinus* | 10_3 |  | 4_9 |  |
| *Charadrius bicinctus* | 1_12 |  |  |  |
| *Charadrius dubius* | 10_3 |  | 4_9 |  |
| *Charadrius leschenaultii* | 10_3 |  | 4_9 |  |
| *Charadrius mongolus* | 10_3 |  | 4_9 |  |
| *Charadrius placidus* | 8_2 |  | 3_7 |  |
| *Charadrius veredus* | 9_2 |  | 3_8 |  |
| *Gallinago gallinago* | 9_4 |  | 5_8 |  |
| *Gallinago hardwickii* | 10_3 |  | 4_7 | 8_9 |
| *Gallinago megala* | 12_3 |  | 4_7 | 8_11 |
| *Gallinago nemoricola* | 11_2 |  | 5_8 |  |
| *Gallinago solitaria* | 10_5 |  | 6_9 |  |
| *Gallinago stenura* | 10_1 | 2_4 | 5_7 | 8_9 |
| *Glareola maldivarum* | 11_3 |  | 4_10 |  |
| *Haematopus ostralegus* | 1_12 |  |  |  |
| *Himantopus himantopus* | 10_3 |  | 4_9 |  |
| *Himantopus leucocephalus* | 1_12 |  |  |  |
| *Hydrophasianus chirurgus* | 9_5 |  | 6_8 |  |
| *Limnodromus semipalmatus* | 10_3 |  | 4_9 |  |
| *Limosa lapponica* | 10_4 |  | 5_9 |  |
| *Limosa limosa* | 10_4 |  | 5_9 |  |
| *Lymnocryptes minimus* | 10_4 |  | 5_9 |  |
| *Numenius arquata* | 8_3 |  | 4_7 |  |
| *Numenius madagascariensis* | 9_2 |  | 3_8 |  |
| *Numenius minutus* | 9_3 |  | 4_8 |  |
| *Numenius phaeopus* | 10_3 |  | 4_9 |  |
| *Phalaropus lobatus* | 11_2 | 3_4 | 5_7 | 8_10 |
| *Pluvialis fulva* | 11_4 |  | 5_10 |  |
| *Pluvialis squatarola* | 11_4 |  | 5_10 |  |
| *Recurvirostra avosetta* | 8_3 |  | 4_7 |  |
| *Rostratula benghalensis* | 11_7 |  | 8_10 |  |
| *Scolopax rusticola* | 10_3 |  | 3_9 |  |
| *Stiltia isabella* | 2_5 |  | 6_1 |  |
| *Tringa brevipes* | 11_3 |  | 4_10 |  |
| *Tringa erythropus* | 11_2 | 3_4 | 5_7 | 8_10 |
| *Tringa glareola* | 10_4 |  | 5_9 |  |
| *Tringa guttifer* | 10_3 |  | 4_9 |  |
| *Tringa nebularia* | 8_4 |  | 5_7 |  |
| *Tringa ochropus* | 8_4 |  | 5_7 |  |
| *Tringa stagnatilis* | 9_3 |  | 4_8 |  |
| *Tringa totanus* | 8_2 |  | 3_7 |  |
| *Vanellus cinereus* | 9_4 |  | 5_8 |  |
| *Vanellus vanellus* | 10_2 |  | 3_9 |  |
| *Xenus cinereus* | 11_3 |  | 4_10 |  |

Note: Numbers are indicative of month periods of the migratory phases for individual species are split in a year. The split is based on visual inspection of the occurrence data (see figure S1) and literature review of the migration ecology of individual species.
