# Supplementary material for: Mapping wader biodiversity along the East Asian—Australasian flyway
Source: PLoS One. 2019 Jan 25;14(1):e0210552. doi: 10.1371/journal.pone.0210552 (PMC6347144; doi:10.1371/journal.pone.0210552)
Supplement: S3 Table — (DOCX) [file pone.0210552.s004.docx]

S3 Table. Total Area by species as estimated Birdlife Range maps and by MaxEnt modelled output, and the difference using Birdlife range maps to subtract MaxEnt outputs (unit: km^2^)

|  | Birdlife | MaxEnt | Difference (Birdlife - MaxEnt) |
| --- | --- | --- | --- |
| *Actitis hypoleucos* | 32,394,898 | 8,035,221 | 24,359,678 |
| *Arenaria interpres* | 5,856,859 | 989,133 | 4,867,726 |
| *Calidris acuminata* | 19,309,923 | 6,231,076 | 13,078,846 |
| *Calidris alba* | 5,655,530 | 1,109,479 | 4,546,051 |
| *Calidris alpina* | 8,963,012 | 990,428 | 7,972,585 |
| *Calidris canutus* | 3,463,497 | 540,320 | 2,923,177 |
| *Calidris falcinellus* | 2,856,060 | 1,483,077 | 1,372,983 |
| *Calidris ferruginea* | 7,053,970 | 2,005,574 | 5,048,397 |
| *Calidris ptilocnemis* | 1,224,904 | 127,958 | 1,096,946 |
| *Calidris ruficollis* | 15,342,000 | 2,254,690 | 13,087,310 |
| *Calidris subminuta* | 3,626,329 | 4,338,132 | -711,803 |
| *Calidris temminckii* | 8,518,483 | 11,449,514 | -2,931,031 |
| *Calidris tenuirostris* | 6,100,433 | 345,328 | 5,755,105 |
| *Charadrius alexandrinus* | 9,211,233 | 1,700,213 | 7,511,020 |
| *Charadrius bicinctus* | 1,699,694 | 302,177 | 1,397,517 |
| *Charadrius dubius* | 23,239,700 | 4,592,340 | 18,647,361 |
| *Charadrius leschenaultii* | 6,707,746 | 780,619 | 5,927,127 |
| *Charadrius mongolus* | 6,793,410 | 1,651,549 | 5,141,861 |
| *Charadrius placidus* | 7,148,333 | 2,557,056 | 4,591,277 |
| *Charadrius veredus* | 5,724,641 | 16,946,456 | -11,221,815 |
| *Gallinago gallinago* | 26,846,447 | 8,091,339 | 18,755,107 |
| *Gallinago hardwickii* | 1,866,372 | 3,072,442 | -1,206,070 |
| *Gallinago megala* | 8,966,958 | 13,201,869 | -4,234,911 |
| *Gallinago nemoricola* | 1,492,459 | 45,848 | 1,446,611 |
| *Gallinago solitaria* | 9,365,553 | 5,779,371 | 3,586,182 |
| *Gallinago stenura* | 22,882,981 | 9,725,913 | 13,157,067 |
| *Glareola maldivarum* | 15,085,678 | 4,108,001 | 10,977,677 |
| *Haematopus ostralegus* | 3,120,741 | 231,209 | 2,889,532 |
| *Himantopus himantopus* | 23,467,868 | 5,247,857 | 18,220,010 |
| *Hydrophasianus chirurgus* | 5,766,020 | 1,590,916 | 4,175,105 |
| *Limnodromus semipalmatus* | 3,681,095 | 1,098,124 | 2,582,971 |
| *Limosa lapponica* | 7,438,845 | 703,897 | 6,734,948 |
| *Limosa limosa* | 18,410,776 | 3,732,051 | 14,678,725 |
| *Lymnocryptes minimus* | 9,093,116 | 2,186,056 | 6,907,060 |
| *Numenius arquata* | 16,506,705 | 6,615,163 | 9,891,542 |
| *Numenius madagascariensis* | 12,940,887 | 421,284 | 12,519,602 |
| *Numenius minutus* | 3,637,729 | 6,490,036 | -2,852,307 |
| *Numenius phaeopus* | 7,132,101 | 1,052,886 | 6,079,215 |
| *Phalaropus lobatus* | 11,458,027 | 13,486,984 | -2,028,957 |
| *Pluvialis fulva* | 11,194,056 | 2,131,043 | 9,063,013 |
| *Pluvialis squatarola* | 11,765,576 | 1,051,957 | 10,713,619 |
| *Recurvirostra avosetta* | 7,006,067 | 7,294,447 | -288,381 |
| *Rostratula benghalensis* | 7,650,768 | 1,047,853 | 6,602,915 |
| *Scolopax rusticola* | 14,931,493 | 3,698,620 | 11,232,872 |
| *Stiltia isabella* | 5,012,499 | 3,698,620 | 1,313,878 |
| *Tringa brevipes* | 8,562,615 | 1,196,291 | 7,366,325 |
| *Tringa erythropus* | 8,910,325 | 10,806,759 | -1,896,435 |
| *Tringa glareola* | 26,251,226 | 7,641,773 | 18,609,454 |
| *Tringa guttifer* | 612,845 | 256,137 | 356,709 |
| *Tringa nebularia* | 24,209,872 | 4,865,478 | 19,344,394 |
| *Tringa ochropus* | 20,592,285 | 6,016,164 | 14,576,121 |
| *Tringa stagnatilis* | 9,670,202 | 4,550,623 | 5,119,579 |
| *Tringa totanus* | 16,765,897 | 3,504,410 | 13,261,487 |
| *Vanellus cinereus* | 6,629,836 | 1,441,826 | 5,188,010 |
| *Vanellus vanellus* | 11,872,199 | 3,940,816 | 7,931,383 |
| *Xenus cinereus* | 15,955,713 | 1,549,084 | 14,406,629 |
