# Supplementary figures and images for: Mapping wader biodiversity along the East Asian—Australasian flyway
Source: PLoS One. 2019 Jan 25;14(1):e0210552. doi: 10.1371/journal.pone.0210552 (PMC6347144; doi:10.1371/journal.pone.0210552)

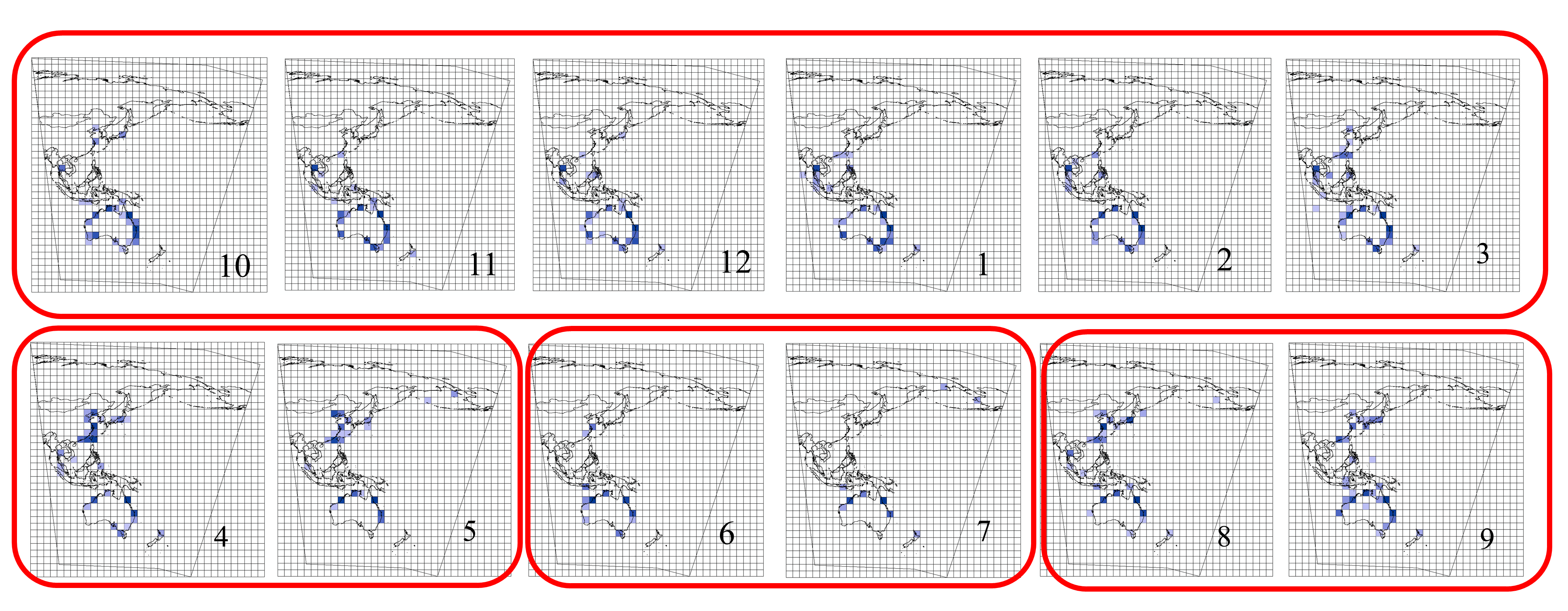

Supplement: S1 Fig — Top: Non-breeding period—Oct, Nov, Dec, Jan, Feb, March; Bottom left: Northward migration—April, May; Bottom middle: Breeding period—June, July; Bottom right: southward migration—August, September. (TIF) [file pone.0210552.s005.tif]

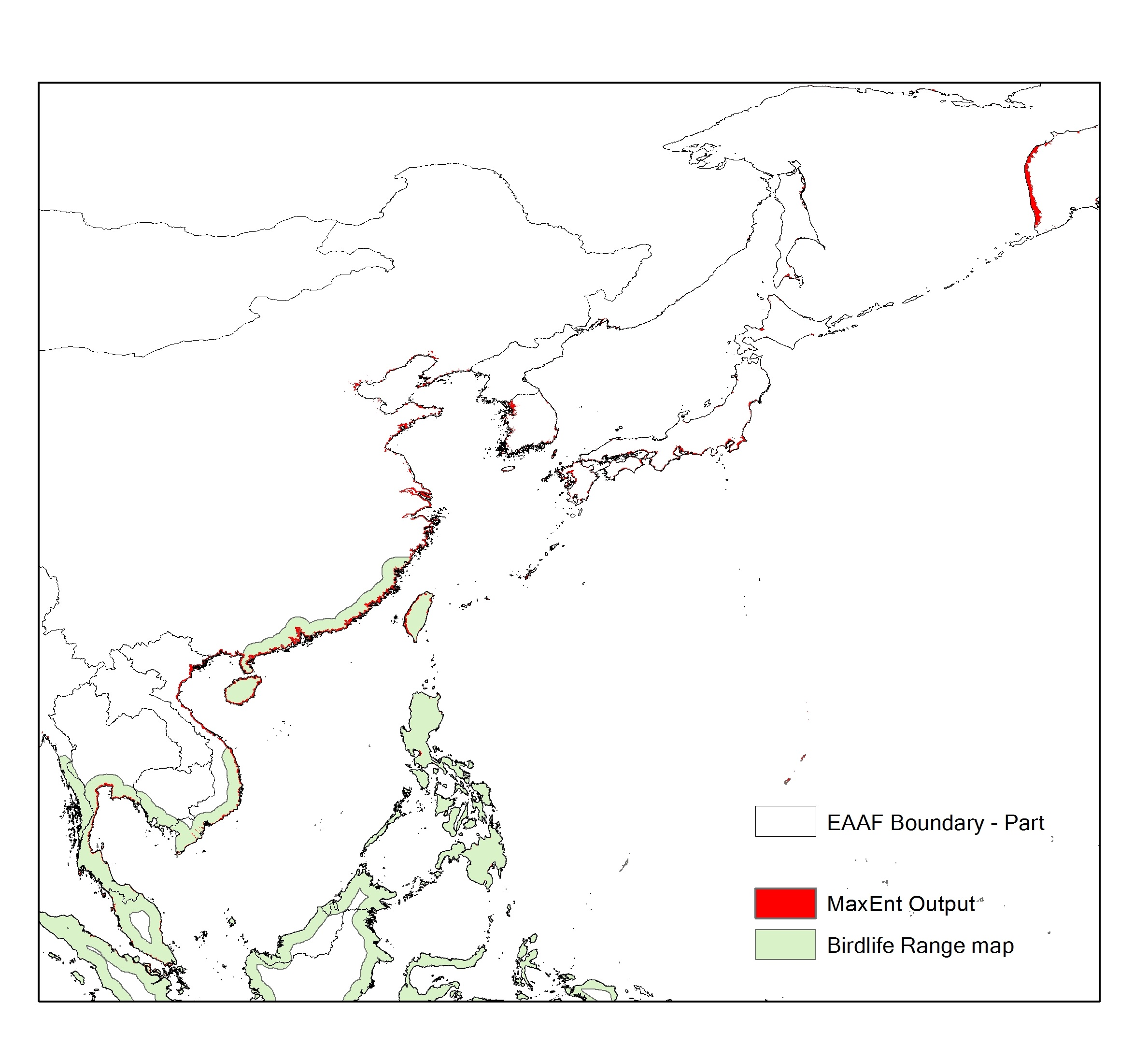

Supplement: S2 Fig — (TIF) [file pone.0210552.s006.tif]
